# Brain structural and functional dissociated patterns in schizophrenia

**DOI:** 10.1186/s12888-017-1194-5

**Published:** 2017-01-31

**Authors:** Chuanjun Zhuo, Jiajia Zhu, Chunli Wang, Hongru Qu, Xiaolei Ma, Hongjun Tian, Mei Liu, Wen Qin

**Affiliations:** 1 Department of Radiology and Tianjin Key Laboratory of Functional Imaging, Tianjin Medical University General Hospital, No. 154, Anshan Road, Heping District, Tianjin, 300052 China; 2 Department of Psychiatry Functional Neuroimaging Laboratory, Tianjin Mental Health Center, Tianjin Anding Hospital, Tianjin, China; 3 Department of Psychiatry, Tianjin Anning Hospital, Tianjin, 300300 China; 4 Department of Psychiatry, Wenzhou Seventh People’s Hospital, Wenzhou, Zhejiang Province 325000 China

**Keywords:** Schizophrenia, Functional connectivity, Gray matter volume, Spatial distribution, Structural brain alterations

## Abstract

**Background:**

Although previous studies found that aberrations in gray matter volume (GMV) and global functional connectivity density (gFCD) are important characteristics of schizophrenia, to the best of our knowledge no study to date has investigated the associations between the spatial distribution patterns of GMV and gFCD alterations. We investigated pattern changes in gFCD and GMV among patients with schizophrenia and their associated spatial distributions.

**Methods:**

Ninety-five patients with schizophrenia and 93 matched healthy controls underwent structural and resting-state functional MRI scanning to assess gFCD and GMV.

**Results:**

We found that gFCD increased in the subcortical regions (caudate, pallidum, putamen, and thalami) and limbic system (left hippocampus and parahippocampus), and decreased in the posterior parieto-occipito-temporal cortices (postcentral gyri, occipital cortex, temporo-occipital conjunction, and inferior parietal lobule), in patients with schizophrenia. By contrast, we found decreased GMV in brain regions including the frontal, parietal, temporal, occipital, cingulate cortices, and the insular, striatum, thalamus in these patients. Increased gFCD primarily occurred in subcortical regions including the basal ganglia and some regions of the limbic system. Decreased gFCD appeared primarily in the cortical regions. There were no statistically significant correlations between changes in gFCD and GMV, and their spatial distribution patterns, in different regions.

**Conclusions:**

Our findings indicate that gFCD and GMV are both perturbed in multiple brain regions in schizophrenia. gFCD and GMV consistently decreased in the cortical regions, with the exception of the Supplementary Motor Area (SMA). However, in the sub-cortical regions, the alterations of gFCD and GMV showed the opposite pattern, with increased gFCD and decreased GMV simultaneously observed in these regions. Overall, our findings suggest that structural and functional alterations appear to contribute independently to the neurobiology of schizophrenia.

## Background

In the past few decades, numerous MRI studies have reported that schizophrenia is associated with widespread brain structural and functional alterations [1–14]. Previous studies have consistently confirmed that patients with schizophrenia have a diffused gray matter volume (GMV) decreases and that this is predominantly located in various cortical and subcortical brain regions. The overwhelming majority of studies have demonstrated that the frontal, temporal, and parietal lobes, cingulate cortex, hippocampus, thalami, and basal ganglia are key regions of GMV decrease in schizophrenia [1–8].

Echoing the alterations in brain structure in schizophrenia, considerable fMRI evidence suggests that functional alterations characterized by hyper-functional activity in sub-cortical regions (mainly in the basal ganglia and some regions of limbic system) with coexistent hypo- or hyper-functional activity in cortical regions occur in schizophrenia. The frontal, temporal and parietal lobes, cingulate gyrus, occipital gyrus, hippocampus, and thalami are key regions of functional alteration in schizophrenia [9–20].

According to previous studies, whole-brain measurement (VBM) is an unbiased and fully automated method used to investigate GMV alterations, which can be used as structural biomarkers for clinical and research applications in psychiatric disorders [21, 22]. Functional connectivity density mapping (FCDM) is a voxel-wise data-driven method that measures the number of functional connections between a given voxel and other voxels in the whole brain, producing a measure of global functional connectivity density (gFCD) [23–26]. gFCD has been developed to measure the number of resting-state functional connections of a given voxel with all other voxels in the entire brain and reflects a one-to-many relationship. While traditional functional connectivity mainly measures the connectivity (FC) strength between two voxels or regions or networks, which reflects a one-to-one relationship [23–27, 28]. Compared to traditional FC, gFCD can reflect the functional activity aberrant from another perspectives, a recent study have shown that gFCD is also a biomarker for psychiatric disorders [26]. Although previous studies found that aberrations in GMV and gFCD are important characteristics of schizophrenia, to the best of our knowledge no study to date has investigated the associations between the spatial distribution patterns of GMV and gFCD alterations.

Therefore, in the present study we aimed to investigate the alterations in GMV and gFCD, and their spatial distribution patterns in patients with schizophrenia, using VBM and FCDM. Several previous studies [17, 29, 30] reported that anatomical and functional brain aberrant are significantly dissociated with schizophrenia, anatomical aberrant has been most robust within thalamo-cortical regions, while functional aberrant has been most pronounced in fronto-parietal and default-mode networks regions. More importantly, the spatial and temporal distributions of anatomical and functional alterations are uncorrelated and the aberrant patterns are also inconsistent or even contradictory, demonstrate a “distinct patterns” [17]. Based on these findings, we hypothesized that gFCD and GMV would be altered in patients with schizophrenia compared with matched healthy controls. We also hypothesized that gFCD and GMV would both be affected in particular brain regions but that their spatial distributions and aberrant patterns (increased or decreased) are also unrelated, or even more complex [31–33].

## Methods

### Subjects

**Fig. 1:**
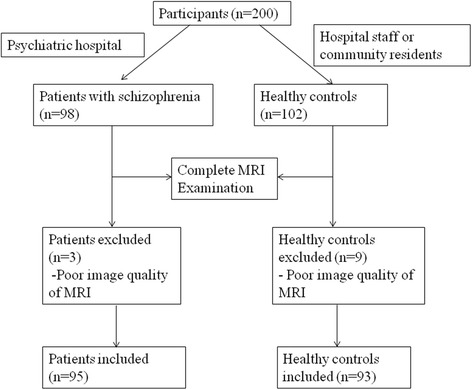
Flow chart of the participants

A total of 200 right-handed subjects participated in the study, including 98 patients with schizophrenia and 102 healthy controls. The patients were recruited from Tianjin Aning Hospital and Tianjn Anding Hospital and the healthy controls were recruited from the hospital staff or community residents. Patient diagnoses and illness duration were determined by the consensus of two senior psychiatrists using the Structured Interview for DSM-IV Axis I Disorder (SCID). All healthy controls were screened using the non-patient edition of the SCID to confirm a lifetime absence of psychiatric illnesses. Healthy controls were interviewed to exclude individuals with a known history of psychiatric illness in first-degree relatives. Exclusion criteria for all subjects were a history of head trauma with consciousness disturbances lasting more than 5 min, a history of substance abuse, pregnancy, or any physical illness, such as cardiovascular disease and neurological disorders, as diagnosed by interview or a review of their medical records. A professional radiologist assessed the MRIs slice-by-slice, and three patients and nine healthy controls were excluded owing to poor image quality. Consequently, the results for 95 patients with schizophrenia and 93 controls were analyzed (Fig. 1). The Positive and Negative Syndrome Scale (PANSS) was used to assess the severity of patients’ psychotic symptoms. The Medical Research Ethics Committee of Tianjin Medical University General Hospital approved this study. Written informed consent was gained from each participant following a complete description of the study.

### MRI data acquisition

MRI was performed using a 3.0-Tesla MR system (Discovery MR750, General Electric, Milwaukee, WI, USA). Tight, but comfortable, foam padding was used to minimize head motion, and earplugs were used to reduce scanner noise. Sagittal 3D T1-weighted images were acquired using a brain volume sequence with the following parameters: repetition time (TR) = 8.2 ms; echo time (TE) = 3.2 ms; inversion time (TI) = 450 ms; flip angle (FA) = 12°; field of view (FOV) = 256 mm × 256 mm; matrix = 256 × 256; slice thickness = 1 mm, no gap; and 188 sagittal slices). Resting-state fMRI data were acquired using a gradient-echo single-short Echo planar imaging sequence with the following parameters: TR/TE = 2000/45 ms; FOV = 220 mm × 220 mm; matrix = 64 × 64; FA = 90°; slice thickness = 4 mm; gap = 0.5 mm; 32 interleaved transverse slices; and 180 volumes. All subjects were requested to keep their eyes closed, relax, move as little as possible, think of nothing in particular, and not fall asleep during the scanning period.

### fMRI data preprocessing

Resting-state fMRI data were preprocessed using Statistical Parametric Mapping software (SPM8; http://www.fil.ion.ucl.ac.uk/spm). The first 10 volumes for each subject were discarded to allow the signal to reach equilibrium and the participants to adapt to the scanning noise. The remaining volumes were corrected for the acquisition time delay between slices. Realignment was then performed to correct for motion between time points. All subjects’ fMRI data were within defined motion thresholds (i.e., translational or rotational motion parameters less than 2 mm or 2°). We also calculated frame-wise displacement, which indexes volume-to-volume changes in head position. Several nuisance covariates (six motion parameters, their first time derivations, and average BOLD signals of the ventricular and white matter) were regressed from the data. Recent study have reported that the signal spike caused by head motion significantly contaminates the final resting-state fMRI results even after regressing out the six motion parameters [34]. Therefore, we further regressed out spike volumes when the frame-wise displacement of the specific volume exceeded 0.5. The datasets were then band-pass filtered in a frequency range of 0.01 to 0.08 Hz [35]. In the normalization step, individual structural images were linearly co-registered with the mean functional image, and then linearly co-registered to the Montreal Neurological Institute (MNI) space. Finally, each filtered functional volume was spatially normalized to the MNI space using the co-registration parameters and were resampled into a 3-mm cubic voxel.

### GMV calculation

The GMV of each voxel was calculated using SPM8 (http://www.fil.ion.ucl.ac.uk/spm/software/spm8/). Structural MR images were segmented into gray matter (GM), white matter and cerebrospinal fluid using the standard unified segmentation model. After an initial affine registration of the GM concentration map into the MNI space, GM concentration images were nonlinearly warped using the diffeomorphic anatomical registration through exponentiated lie algebra technique, and the results resampled to a voxel size of 3 mm × 3 mm × 3 mm. The GMV of each voxel was obtained by multiplying the GM concentration map by the non-linear determinants derived from the spatial normalization step. Finally, the GMV images were smoothed using a Gaussian kernel of 6 mm × 6 mm × 6 mm full-width at half maximum. After spatial preprocessing, the smoothed GMV maps were used for statistical analyses.

### FCD calculation

The FCD of each voxel was calculated using an in-house Linux script according to the method described by Tomasi and Volkow [23–26]. Pearson’s linear correlation evaluated the strength of the functional connectivity between voxels [36, 37]. According to the most of the previous studies, voxel pairs with a correlation coefficient of *R* > 0.6 were considered significantly connected, thereafter, in current study, we also adopted *R* > 0.6 as the cut-off point. [23–26, 38–40], FCD calculations were restricted to the cerebral gray matter mask regions. The gFCD at a given voxel, x_0_, was computed as the global number of functional connections, k(x_0_), between x_0_ and all other voxels. This calculation was repeated for all x_0_ voxels in the brain. The FCD maps were spatially smoothed using a 6 mm × 6 mm × 6 mm Gaussian kernel to minimize differences in the functional brain anatomy across subjects.

### Statistical analysis

Group differences in GMV and gFCD were compared in a voxel-wise manner using a two-sample *t*-test with age and sex as nuisance variables. Multiple comparisons were corrected using a false discovery rate method with a corrected threshold of *P* < 0.05.

The mean gFCD and GMV values of each cluster, with significant group differences in gFCD, were extracted for each subject and used for region of interest (ROI)-based group comparisons. To clarify if the regions with altered gFCD also showed structural impairment, we first tested the group differences in GMV for each ROI using two-sample t-tests after controlling for age and sex. The group differences in gFCD were then tested using two-sample t-tests, after further regressing out the GMV influences, to investigate their separate contributions to functional aberrances in schizophrenia. Finally, a partial correlation coefficient was used to test the relationship between gFCD and GMV in each group, and to test the associations between gFCD and clinical variables, including antipsychotic dose (chlorpromazine equivalents), illness duration, and PANSS scores. Age and sex effects were regressed out and multiple comparisons were corrected using the Bonferroni method (*P* < 0.05).

## Results

### Subject demographics and clinical characteristics

The demographic and clinical characteristics of the subjects are summarized in Table 1. There were no significant group differences in sex (*χ*2 = 1.35, *P* = 0.25) or age (t = 0.48, *P* = 0.63). Eighty-seven patients received medication during the MRI examinations, with the remaining eight patients being treatment naïve. The mean antipsychotic dose (cholorpromazin equivalents) was 446.5 ± 341.6 mg/d for the patients with schizophrenia. The mean duration of illness was 121.4 ± 92.8 months. Mean scores on the PANSS positive, negative, and general psychopathology subscales were 17.1 ± 7.9, 20.3 ± 9.1, and 34.1 ± 10.8, respectively.

**Table 1:** Demographic and clinical characteristics of the participants

Characteristics	Schizophrenia Patients	Healthy Controls	*P* value
Number of subjects	95	93	NA
Age (years)	33.6 (7.8)	33.0 (10.2)	0.633
Gender (female/male)	41/54	48/45	0.246
Antipsychotic dosage (mg/d) (chlorpromazine equivalents)	446.5 (341.6)	–	NA
Duration of illness (months)	121.4 (92.8)		NA
PANSS		–	NA
Positive score	17.1 (7.9)	–	
Negative score	20.3 (9.1)	–	
General psychopathologyscore	34.1 (10.8)	–	
Total score	71.5 (23.2)	–	

Data are shown as the mean (standard deviation). *PANSS* The Positive and Negative Syndrome Scale

### Distributed specificity of gFCD hubs in patients and controls

**Fig. 2:**
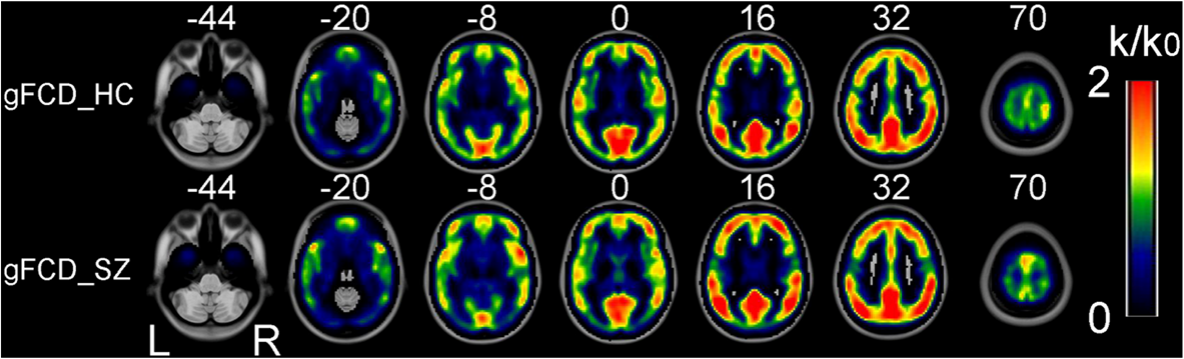
Spatial distributions of the average rescaled gFCD hubs between patients with schizophrenia and controls. Abbreviations: gFCD, global FCD; HC, healthy controls; SZ, patients with schizophrenia

Our results revealed that the strongest gFCD hubs were consistently located in the precuneus, inferior parietal lobe, superior temporal gyrus, medial prefrontal cortex, and dorsal lateral prefrontal cortex in our two groups. Most of the gFCD hubs are located in the default mode network and sensory cortices (Fig. 2). The distribution of the gFCD hubs did not differ between the two groups and was also consistent with the results of Tomasi et al. [24].

### gFCD changes in patients with schizophrenia

**Fig. 3:**
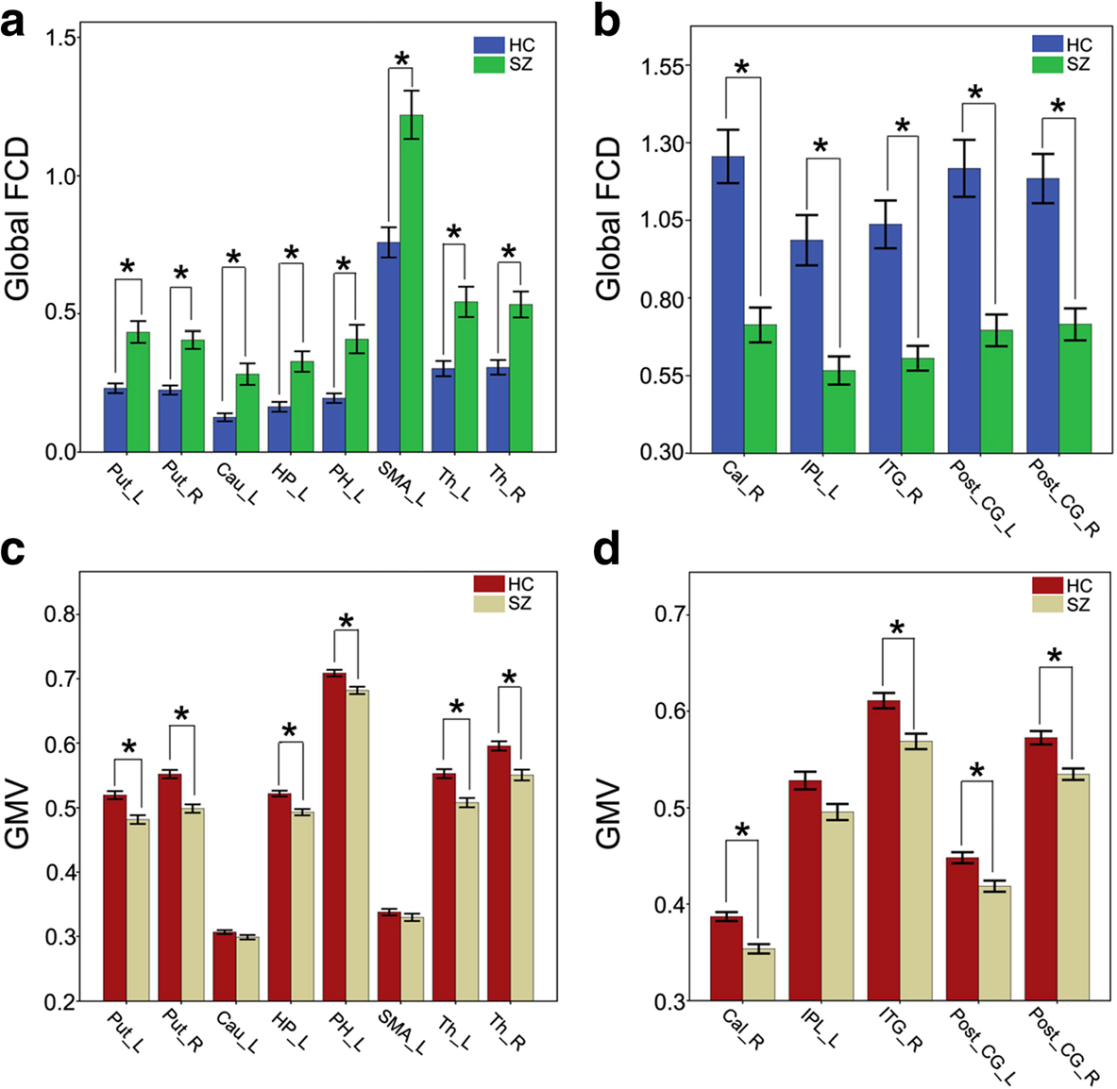
ROI-based gFCD and GMV comparisons in regions exhibiting group differences in gFCD voxel-based analysis. Note: Error bars indicate the standard deviations of the means. *P < 0.05, Bonferroni corrected. Cal, calcarine gyrus; Cau, caudate; FCD, functional connectivity density; HC, healthy controls; HP, hippocampus; IPL, parietal inferior lobule; ITG, inferior temporal gyrus; PH, parahippocampal gyrus; Post CG, postcentral gyrus; Put, putamen; SMA, supplementary motor area; SZ, patients with schizophrenia; Th, thalamus. (**a**) Schizophrenia patient's gFCD is increased compareded to healthy controls in sub-cortical regions (with an exception of supplementary motor area) (**b**) Schizophrenia patient's gFCD is decreased compareded to healthy controls in cortical regions (**c**) Schizophrenia patient's GMV is decreased compareded to healthy controls in sub-cortical regions (**d**) Schizophrenia patient's GMV is decreased compareded to healthy controls in cortical regions

Voxel-wise analysis showed increased gFCD in the sub-cortical and limbic system areas (including the bilateral putamen, caudate, pallidum, and thalami), the hippocampal/parahippocampal complex, and the supplementary motor area (SMA) (Fig. 3a, Table 2). Decreased gFCD was seen in the posterior cortices, including the bilateral postcentral gyri, precentral gyri, right calcarine sulcus, and the left inferior occipital gyrus lobule in patients with schizophrenia (Fig. 3b, Table 2). ROI-based analyses showed that the differences in gFCD between schizophrenia patients and controls persisted even further regress out the GMV effect, suggesting that gFCD alterations in these ROIs are relatively independent characteristics of schizophrenia.

**Table 2:** Global FCD changes in patients with schizophrenia relative to healthy controls

Regions	Brodmann areas	Cluster size (Voxels)	Peak t values	Coordinates in MNI (x, y, z)
Schizophrenia patients > Healthy controls				
Left putamen	–	218	4.87	−12, 3, −6
Right putamen	–	177	5.09	12, 12, −9
Left caudate	–	77	3.75	−15, −18, 18
Left hippocampus	–	59	4.35	−27, −33, −6
Left parahippocampal gyrus	–	47	4.47	−27, −24, −18
Left supplementary motor area	6	28	4.52	−3, 0, 72
Left thalamus	–	31	3.94	−6, −27, 0
Right thalamus	–	42	4.18	9, −24, −3
Schizophrenia patients < Healthy controls				
Right calcarine gyrus	18	132	−4.67	18, −87, 12
Left parietal inferior lobule	40	31	−4.12	−39, −33, 36
Right inferior temporal gyrus	37	46	−4.67	42, −66, −6
Left postcentral gyrus	4, 6	110	−4.81	−54, −12, 36
Right postcentral gyrus	2, 3, 4	90	−4.32	48, −24, 45

*FCD* functional connectivity density, *MNI* Montreal Neurological Institute

### Gray matter volume changes in patients with schizophrenia

Decreased GMV was seen only in the bilateral frontal, parietal, temporal, occipital lobule, cingulate cortex, insular, striatum and thalamus in patients with schizophrenia (Fig. 3c and d and Table 3).

**Table 3:** Group differences in GMV between patients with schizophrenia and controls

	HC	SZ	F	Sig.
Striatum_L	0.520 ± 0.059	0.482 ± 0.066	17.778	<0.001
Striatum_R	0.552 ± 0.062	0.499 ± 0.064	35.410	<0.001
Cau_L	0.306 ± 0.030	0.299 ± 0.034	2.436	0.120
HP_L	0.522 ± 0.043	0.493 ± 0.048	17.063	<0.001
PH_L	0.709 ± 0.049	0.682 ± 0.055	11.519	0.001
SMA_L	0.338 ± 0.049	0.330 ± 0.056	.726	0.395
Th_L	0.553 ± 0.067	0.508 ± 0.071	17.739	<0.001
Th_R	0.596 ± 0.070	0.551 ± 0.082	13.913	<0.001
Cal_R	0.387 ± 0.045	0.353 ± 0.047	22.458	<0.001
IPL_L	0.528 ± 0.089	0.496 ± 0.082	5.438	0.021
ITG_R	0.611 ± 0.077	0.569 ± 0.078	14.333	<0.001
Post_CG_L	0.448 ± 0.055	0.419 ± 0.056	11.270	0.001
Post_CG_R	0.572 ± 0.067	0.535 ± 0.058	14.566	<0.001

age and sex effects were regressed out

### Associations between the spatial distributions of gFCD and GMV

As previously mentioned, increased gFCD were primarily located in subcortical regions, including the basal ganglia and some components of the limbic system and SMA. Decreased gFCD were primarily located in the posterior cortical regions. However, in this study, only decreased GMV was observed. Notably, the aberrant patterns of gFCD and GMV differ between regions. In the caudate, thalami and hippocampus complex (HPC), gFCD increased but GMV decreased, while in the posterior cortices, decreased gFCD accompanied decreased GMV. These findings indicated a complex association between gFCD aberrances and GMV changes in schizophrenia. To further investigate whether gFCD correlates with GMV changes, we also performed correlation. In multiple regions, we found that although gFCD and GMV were both affected, there was no statistical correlation between gFCD and GMV either in patients with schizophrenia patients or controls (Fig. 3).

### Association between gFCD/GMV and clinical variables

We did not find any statistical correlation between gFCD or GMV and antipsychotic dose, illness duration, or any PANSS scores (negative, positive, general psychopathology, or total scores).

## Discussion

In this study, we first used voxel-wise graph theory to investigate the spatial distribution patterns of gFCD and GMV alterations in a relatively large schizophrenic patient data set. We found that gFCD and GMV were both affected in some regions of the basal ganglia, thalami, limbic system, and posterior cortices, which are the key regions contributing to the pathology of schizophrenia as indicated by several lines of evidence.

More importantly, in our findings, decreased gFCD was found in areas primarily located in the posterior cortical regions, while increased gFCD was observed primarily in areas located in the sub-cortical and limbic system regions. However, only decreased GMV was found in either the cortical or sub-cortical regions in patients with schizophrenia.

In our previous study, we demonstrated for the first time that the gFCD aberrant in schizophrenia, we found increased gFCD in the bilateral striatum and hippocampus and decreased rsFCD in the bilateral sensorimotor cortices and right occipital cortex [27]. Our previous findings added new evidence for supporting the hypothesis that schizophrenia is a connectivity disorder from the perspective of functional connectivity density [27]. And our findings had been confirmed by the subsequent studies [41–43].

As previously mentioned, some cortical regions (e.g., the frontal, temporal, and parietal lobes, cingulate gyrus, cuneus, and others), limbic system (e.g., hippocampus, parahippocampus, and thalami) and basal ganglia are key regions of functional connectivity and GMV aberrances in schizophrenia. In this study, gFCD and GMV were both affected in most of these regions, but the spatial distribution pattern of gFCD and GMV differed between regions. With the exception of the SMA, in the cortical regions, gFCD and GMV consistently decreased. However, in the sub-cortical regions, the alterations in gFCD and GMV contrasted, with increased gFCD and decreased GMV simultaneously observed in these regions.

Our results suggest that the association between anatomical changes and functional activity aberrances is complex and undefined. Previous studies also demonstrated that structural deficits do not have a straightforward relationship with functional abnormalities [20, 44, 45]. Thus, longitudinal studies aiming to pinpoint the onset of connectivity abnormalities as schizophrenia develops will help clarify the nature of the associations between the structural deficits and functional connectivity alterations in schizophrenia [44].

In this study, we did not found a correlation between aberrant of gFCD/GMV and antipsychotic dose, illness duration, and symptom severity, this finding is consistent with previous studies. and suggest that illness duration, symptom severity, and antipsychotic dose are not linearly related to brain functional or structural abrreant [17, 32, 41, 44]. Our findings suggested that aberrant gFCD and GMV maybe an independent trait characteristic of schizophrenia. Our findings also support the hypothesis that BOLD signal alterations may better track the presence of psychosis rather than its severity or duration [20], and is not linearly related to the antipsychotic dose [46].

## Conclusion

This study provides new evidence of aberrant gFCD and decreased GMV in multiple brain regions contributing to the pathology of schizophrenia in a relatively large sample of patients with schizophrenia. More importantly, we revealed that gFCD and GMV are jointly affected in multiple regions, but that their spatial distribution patterns in the intergroup difference maps were dissociated. These findings suggest that structural and functional alterations probably contribute independently to the neurobiology of schizophrenia. However, the nature of the associations between the structural deficits and functional alterations needs clarification using longitudinal studies to pinpoint the onset of connectivity abnormalities as schizophrenia develops.

## Abbreviations

FCD: Functional connectivity density
gFCD: Global functional connectivity density
GMV: Gray matter volume
PANSS: Positive and negative syndrome scale
SCID: Structural clinical interview for DSM-IV
